# One Shoe Doesn't Fit All: A Contextual Approach to Therapeutic Footwear for People at Risk of Diabetes‐Related Foot Ulceration

**DOI:** 10.1002/jfa2.70176

**Published:** 2026-06-17

**Authors:** Gustav Jarl, Peter A. Lazzarini, Gulapar Srisawasdi, Jaap J. van Netten

**Affiliations:** ^1^ Department of Prosthetics and Orthotics Faculty of Medicine and Health Örebro University Örebro Sweden; ^2^ University Health Care Research Center Faculty of Medicine and Health Örebro University Örebro Sweden; ^3^ Allied Health Research Collaborative The Prince Charles Hospital Brisbane Australia; ^4^ School of Public Health and Social Work Queensland University of Technology Brisbane Australia; ^5^ Department of Rehabilitation Medicine Faculty of Medicine Siriraj Hospital Mahidol University Bangkok Thailand; ^6^ Sirindhorn School of Prosthetics and Orthotics Faculty of Medicine Siriraj Hospital Mahidol University Bangkok Thailand; ^7^ Department of Rehabilitation Medicine Amsterdam UMC University of Amsterdam Amsterdam the Netherlands; ^8^ Amsterdam Movement Sciences, Rehabilitation & Development Amsterdam the Netherlands

**Keywords:** diabetes complications, diabetic foot, diabetic neuropathies, footwear, orthotic devices, patient compliance, shoes, treatment adherence and compliance

## Abstract

**Introduction:**

People at risk of diabetes‐related foot ulcers are recommended to always use therapeutic footwear when weight‐bearing, to help prevent ulcers. These recommendations are supported by good quality evidence, yet adherence by patients to using this footwear is low. One reason may be because these recommendations do not consider that footwear use is highly contextual to the physical or sociocultural environments it is intended to be used. In this paper, we propose and discuss a contextual approach to considering therapeutic footwear solutions.

**Contextual Approach to Therapeutic Footwear:**

Recommending patients to use the same therapeutic footwear solution in vastly different contexts seems at odds with person‐centred care principles and is likely a reason for patients not fully adhering to such recommendations. We discuss seven contexts in which using therapeutic footwear is particularly challenging: the home, workplaces, social occasions, places‐of‐worship, water‐related activities, hotter climates and holidays. We outline how a contextual approach to therapeutic footwear might lead to more appropriate footwear solutions. This approach typically involves a trade‐off between functional benefits and adherence and may lead to novel designs for different contexts. We also propose six different footwear solutions that incorporate features that may be more aesthetically pleasing, cooler, lighter, cheaper or waterproof, yet still providing protection and functional offloading. We suggest outcomes of such an approach may be more favourable for the patient's overall adherence and foot health than the current somewhat unrealistic recommended practice of prescribing a “one shoe fits all” solution.

**Conclusion:**

A contextual approach as proposed in this paper may lead to novel therapeutic footwear solutions that better address patients' needs for adequate footwear. If these footwear solutions are implemented, this may in future lead to higher patient adherence to using appropriate footwear and lower ulceration rates.

## 1 Introduction

Foot ulcers are a common and devastating complication of diabetes, affecting up to 34% of people with diabetes during their lifetime [1]. Whilst the majority of foot ulcers heal, approximately 40% will recur within 1 year [1]. With diabetes‐related foot disease, including foot ulcers, a leading cause of the global disease and disability burden [2, 3], preventative interventions are essential in populations at risk of developing foot ulcers [4].

Diabetes‐related foot ulcers are the outcome of a complex process involving numerous factors [1]. Simplified, the development of foot ulcers can be described as a two‐step process [5]. First, predisposing factors, especially peripheral neuropathy, peripheral artery disease and foot deformities, increase the risk of developing foot ulcers. Second, one or more precipitating factors, typically external mechanical or thermal trauma, trigger the development of foot ulcers [6]. Thus, evidence‐based guidelines recommend people at moderate or high risk of foot ulceration to always use therapeutic footwear when weight‐bearing, to protect the foot from these precipitating factors and in turn reduce the risk of foot ulceration [4].

**FIGURE 1:**
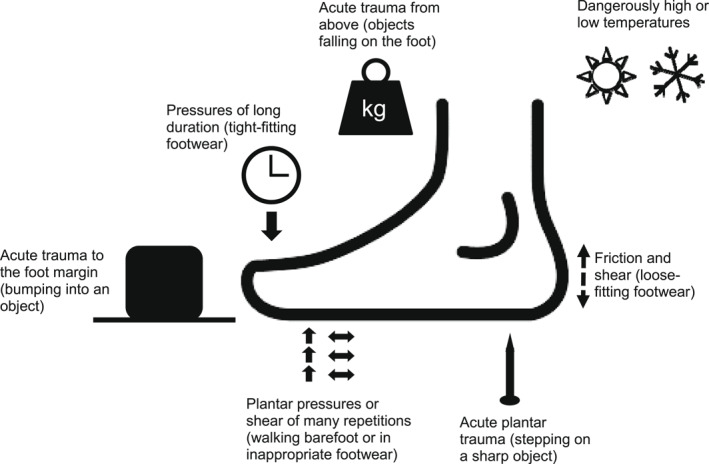
Common acute and chronic trauma that precipitates foot ulcers in people at increased risk. Therapeutic footwear should protect against acute trauma (falling objects, bumping into objects, stepping on sharp objects, extreme temperatures) and chronic trauma (high mechanical pressure, and movement and shear during walking, pressure of long duration). Foot image: Flaticon.com.

It is widely thought fundamental that people with diabetes at‐risk of foot ulceration use therapeutic footwear that protect against both acute and chronic external trauma [4]. As depicted in Figure 1, acute trauma typically affect the dorsal foot (objects falling on the foot), foot margins (bumping the foot into an object), plantar surface (stepping on a sharp object), or the whole foot (extreme temperatures). Chronic trauma typically affects the plantar surface of the foot via repetitive high mechanical pressure and shear during normal walking, which often results in ulceration if not reduced or redistributed by using adequate therapeutic footwear [7]. Further, footwear should not itself cause trauma to the foot that could result in an ulcer. For example, high heels increase forefoot pressures, loose‐fitting footwear may result in movement and shear, and tight‐fitting footwear may result in pressures of long duration [8, 9].

The aim of always protecting the foot from this variety of potential trauma is the basis for recommending therapeutic footwear with a variety of features to be used at all times. Such features include custom‐made insoles, low heel height and rocker‐bottom outsoles to reduce high plantar pressure; wide a deep toe boxes to prevent pressures on the toes; firm heel counters and fastening mechanisms to prevent movement and shear; and appropriate upper coverings and outsoles to protect the foot from acute trauma and insulate against extreme temperatures (Table 1) [4, 10, 11, 12]. However, adherence to using such therapeutic footwear is often much lower than recommended in guidelines [13]. This low adherence creates a ‘double whammy’ type of situation, as the patient not only loses the aforementioned protective features of therapeutic footwear [14], but also ‘gains’ trauma‐inducing features when choosing less appropriate footwear, both increasing the risk for foot ulceration [15].

**TABLE 1:** Frequently recommended features of therapeutic footwear.

Footwear feature	Purpose	Potential disadvantage to patients
Rigid rocker‐bottom outsole	Reduce plantar pressures on metatarsal head region.	May impair balance, adds weight to footwear.
Extra‐depth footwear	Prevent pressure on deformed toes, accommodate custom‐made insole.	Less aesthetically appealing footwear.
Wide toe box	Prevent pressure on the toes.	Less aesthetically appealing footwear.
Low heel height	Reduce plantar pressures on forefoot.	Less aesthetically appealing footwear.
Firm heel counter and retaining mechanism (laces or Velcro strap)	Prevent movement and shear and protect toes from pressure due to slip of the footwear.	Cumbersome to put on and take off footwear, excludes slippers.
High footwear shaft	Reduce forefoot pressure, movement and shear.	Increased footwear weight, too hot in some climates or some periods of the year.
Covered footwear	Protect against acute trauma.	Too hot in some climates or some periods of the year, less aesthetically appealing for some.
Custom‐made footwear and insoles	Accommodate foot deformities and oedema.	Inaccessible in some settings because of high costs and lack of trained footwear providers.

Quantitative studies on footwear adherence have found inconsistent results regarding reasons for footwear non‐adherence [16, 17, 18, 19]. A meta‐synthesis of qualitative studies identified that patients seem to engage in ‘strategic non‐adherence’, where they knowingly deviate from recommended footwear in order to live a more normal daily life [20]. This indicates a tension between the static comprehensive guideline‐based footwear recommendations made for all eventualities, and the different dynamic contexts of patients' daily life. Thus, average footwear adherence rate measures may in fact hide widely differing adherence rates for different specific contexts. For example, one study found lower adherence to using therapeutic footwear at home than away from home, and that the predictors for adherence at home differed from the predictors for adherence away from home [19]. In another study, participants reported that their low adherence to using therapeutic footwear inside the house was because their footwear was heavy and difficult to don and doff, yet the footwear was equally heavy and difficult to don and doff outside the house as well, where adherence was higher [21].

Thus, the ‘one‐(therapeutic) shoe‐fits‐all’ approach as currently recommended, where the same therapeutic footwear is recommended to be used in all contexts, may lead to lower overall patient adherence and more ulcers when compared to recommending different footwear for different contexts. Given the increasing emphasis on personalised medicine in health care and foot care [22], in this paper we aim to propose and discuss a contextual approach to considering therapeutic footwear solutions.

## 2 Challenging Contexts to Using Therapeutic Footwear

People's ‘contexts’, defined here as their physical and sociocultural settings, influence their choice of clothing, including their choice of footwear [23]. Based on expert opinion, that is, clinical experience supported by the literature cited below, we identified seven different contexts in which patients find adherence to using therapeutic footwear particularly challenging, including: the home, workplaces, social occasions, water‐related activities, hotter climates, places‐of‐worship, and holidays (Table 2). We discuss the implications of each of these contexts on adherence to using therapeutic footwear below.

**TABLE 2:** Summary of footwear contexts, adherence barriers, inadequate patient solutions and suggested footwear solutions.

Context	Barriers to adherence	Inadequate patient solutions	Suggested footwear solutions
Home	Patients' impression that home environment is ‘safe’ and little walking takes place there, outdoor footwear not culturally acceptable indoors, outdoor footwear being dirty, inconvenience to put on footwear.	Walking barefoot, in socks without footwear or in slippers.	Easy‐to‐put‐on indoor therapeutic footwear, light‐weight therapeutic sandals covering the toes, padded hosiery, insole‐in‐sock
Workplaces	A. Requirements of safety footwear.	A. Safety footwear with toecaps, without enough space for deformed toes and custom‐made insoles.	Therapeutic safety footwear
	B. Aesthetic requirements.	B. Tight‐fitting, possibly high‐heeled, footwear without space for custom‐made insoles.	Aesthetically pleasing therapeutic footwear
Social occasions	Patients prioritize footwear aesthetics over protective features.	Open, tight‐fitting, possibly high‐heeled, footwear.	Aesthetically pleasing therapeutic footwear
Water‐related activities	Therapeutic footwear cannot be worn in water.	Flip flops, sandals without socks, or walk barefoot.	Waterproof therapeutic footwear
Hotter climates	Closed footwear may cause skin macerations and fungal infections, and is less pleasant to use.	Flip flops, sandals without socks, or walk barefoot outdoors.	Light‐weight therapeutic sandals covering the toes
Places‐of‐worship	Visitors are expected to remove their footwear.	Walk barefoot.	Padded hosiery, insole‐in‐sock
Holidays	Patients out of daily routines, including footwear habits.	Barefoot walking and inappropriate footwear of different types.	Mix of solutions suggested above

### 2.1 Home

Patients perceive their home to be a safe environment, relatively free from external trauma, and in which they take relatively few steps compared with steps away from their home [21]. However, studies have now consistently found that patients on average take more steps in their home than away from their home [21, 24, 25]. These misperceptions may be one reason why adherence to using therapeutic footwear is much lower in the home than away from home [21, 24, 25, 26]. Other reasons for low adherence in the home may be for cultural reasons, out of habit, or that therapeutic footwear typically is heavy (due to materials used to make the outer sole rigid) and often difficult to don and doff [21]. These features are especially challenging for patients who have difficulties reaching their feet, have impaired mobility, hand function, or eyesight [27, 28]. For night‐time visits to the bathroom, patients may have even less tolerance for donning and doffing footwear. As a result, patients frequently walk barefoot, in slippers or in socks without footwear in their home, which exposes their feet to both acute and chronic trauma [29].

### 2.2 Workplaces

In certain workplaces, legislation or employers demand employees use footwear that have specific safety or aesthetic features that may conflict with the protective features of therapeutic footwear [30]. Within many manual labouring jobs, safety regulations typically require that employees use safety footwear. Although standard safety footwear protects against external trauma and insulate against extreme temperatures, they do not have rocker‐bottomed outsoles and often have limited space for incorporating custom‐made insoles, resulting in inadequate plantar pressure reduction. In addition, narrow unforgiving steel toecaps can cause pressure and in turn ulcers on toes [31]. Legislation often also does not allow modifications to be made once safety footwear is delivered, even though improving footwear based on pressure measurements is recommended in guidelines [12]. In other workplaces, employees may be required to use footwear with certain aesthetic attributes that conflict with protection of the feet [30]. For example, flight attendants are sometimes required to use high heel footwear [32]. High heels are known to cause high plantar forefoot pressures, and this kind of footwear typically lacks space for custom‐made insoles and has a narrow toe box that can cause pressures on the toes. In other workplaces, such as on farms or in other agricultural workplaces, people often must work in rubber boots due to the wet and dirty environment. These boots are typically non‐stretchable and non‐adjustable, thereby increasing pressures on toes and causing movement and shear.

### 2.3 Social Occasions

At weddings, funerals, parties and other social occasions, people often prioritize footwear aesthetics over comfort [33]. Footwear for social occasions are typically tight‐fitting, thereby causing pressures on the toes. Furthermore, such footwear does not prevent high plantar pressures as it does not have rocker bottom outsoles, lacks space for custom‐made insoles and may be high heeled [8, 34]. This is a challenging footwear context, especially for females as women's fashionable footwear are usually more dangerous to feet than men's fashionable footwear.

### 2.4 Water‐Related Activities

Weight‐bearing activities where the feet get wet are particularly challenging when it comes to use of therapeutic footwear. People typically are barefoot when at the beach, when walking around the swimming pool, and sometimes when fishing, or non‐therapeutic footwear may be worn instead. However, this exposes the feet to acute trauma (e.g., from sharp objects hidden in the sand or seabed), burns (if the ground is hot) or high plantar pressures [35].

### 2.5 Hotter Climates

In regions with hotter climates it is common to wear flip flops, sandals without socks or walk barefoot outdoors [36, 37, 38]. Barefoot walking exposes the feet to several kinds of mechanical and thermal trauma and to the risk of plantar ulcers and burns. Thin‐soled flip flops and sandals protect the feet more than walking barefoot (e.g., from plantar burns), but do not protect against acute trauma, nor do they adequately reduce plantar pressures [39]. In addition, flip flops with straps can cause ulcers in an insensate foot due to movement and shear [40], and the lack of heel counters or heel straps can enable the sandal to move and be inadvertently worn inappropriately in people with profound loss of sensation due to neuropathy [41]. However, using closed therapeutic footwear in hotter climates can also increase the risk of fungal infections [42] and some studies suggest that increasing the in‐shoe humidity and temperature in itself might increase the risk of ulceration [43, 44].

### 2.6 Places‐of‐Worship

In certain places‐of‐worship, such as temples or mosques, visitors are expected to remove their footwear when entering the building, resulting in an inability to use any (therapeutic) footwear [45]. Thus, patients have to walk barefoot and expose their feet to a variety of acute and chronic external trauma, such as high plantar pressures or risk of burns, if the floor is hot because of sunlight exposure [45]. In addition, certain worshipping or prayer positions can increase prolonged pressure on different surfaces of the feet, such as the lateral malleoli [46].

### 2.7 Holidays

On holidays, patients tend to move away from their normal daily activity routines, meaning that they may put their feet at risk of foot ulcers in a way that they otherwise do not at home, the so called ‘diabetic holiday foot’ [35, 40, 47]. E.g., patients may spend more time than they do in the rest of the year in contexts described above that potentially elevate their risk of trauma or poor adherence (e.g., social occasions, water‐related activities and hotter climates), while they may also suddenly increase their daily walking and other weight‐bearing activities or engage in unaccustomed exercise [35, 40, 47]. In this sense, holidays are a mix of different challenging contexts. Another challenge with holidays is patients postponing to seek medical attention until they return home when a (pre‐) ulcer develops [48]. This is problematic, as delay of referral and treatment have been associated with worse ulcer healing [49, 50]. The diabetic holiday foot may be a growing problem given that international tourism is increasing, especially in low‐ and middle‐income countries where the prevalence of diabetes also is increasing rapidly [51].

## 3 Potential Contextual Approach Solutions to Using Therapeutic Footwear

Although there are numerous contexts to consider when providing therapeutic footwear, there is overlap between contextual demands and therefore also overlap between footwear solutions. Potential therapeutic footwear solutions, including both already existing solutions and future novel solutions, are discussed below and summarised in Table 2.

### 3.1 Therapeutic Indoor Footwear

As discussed above, adherence to wearing therapeutic footwear at home is lower than optimal, partly because it typically is heavy and difficult to don and doff. This may result in patients using slippers or no footwear at all at home. To increase indoor use, footwear should preferably be comfortable and convenient, as many people are not used to wearing footwear at home. In general, footwear with Velcro or zippers is more convenient to don and doff than footwear with laces. Interestingly, some footwear manufacturers have designed shoes whose heel counters are shaped like a shoehorn to facilitate the donning and doffing without using hands. Furthermore, indoor footwear should be lightweight to be more acceptable to patients. However, the footwear still needs to protect against acute trauma and high plantar pressures. Special therapeutic indoor footwear with similar protective offloading qualities as regular therapeutic footwear is a potential solution to increase the use of therapeutic footwear indoors [52]. Studies have reported development of therapeutic indoor footwear and that provision of such footwear increases adherence to using footwear indoors [53, 54, 55]. Other clinics modify conventional slippers or sandals, or prescribe prefabricated therapeutic sandals with custom‐made insoles for patients to use indoors.

### 3.2 Lightweight Therapeutic Sandals

Many patients prefer to use sandals indoors at home and outdoors in hotter climates as they find regular therapeutic footwear to be too hot. Therefore, clinicians may consider prescribing therapeutic sandals, i.e., footwear that has the attributes of therapeutic footwear but leaves the toes and part of the dorsum of the foot unprotected [56, 57]. This makes using footwear cooler in hotter climates, however, the lack of upper protection exposes the dorsum and toes to acute trauma and allows for foreign bodies (e.g., stones) to enter the sandals and potentially cause foot ulcers [58]. In addition, some patients find therapeutic sandals to be unacceptably heavy, because of the outer sole being reinforced to be rigid with rocker bottoms, while in other countries healthcare professionals are not allowed to prescribe open footwear to people at risk of foot ulceration. Future development may include using other materials and designs to reduce the weight and protect the whole foot, while remaining breathable and colder, a development that patients especially see as important [59].

### 3.3 Therapeutic Safety Footwear

Patients with work that demand safety footwear pose a particular challenge to foot ulcer prevention; they often spend most of their daily weight‐bearing activity at work where they are not allowed to use therapeutic footwear. Negotiations with employers are sometimes possible to increase the patient's duties that don't need safety footwear or make exceptions from the demand of safety footwear. However, there is a need for footwear manufacturers to develop therapeutic safety footwear that fulfils both formal safety requirements and protective offloading requirements. Such footwear should include rocker bottom outsoles, have wide and high toecaps that leave room for deformed toes, be overall generous in volume to allow for oedema and custom‐made insoles, and allow for adjustments to be made following plantar pressure measurements and to resolve wear and tear.

### 3.4 Aesthetically Appealing Therapeutic Footwear

Therapeutic footwear often deviates from aesthetic ideals for conventional footwear. This may lead to patients rejecting the footwear and instead wear conventional footwear which provide inadequate protection for the foot. Although it may be challenging to change the shoe last to improve footwear appearance, while still retaining plantar pressure offloading properties and accommodating the shape of one's foot, it may be feasible to design more aesthetically pleasing therapeutic footwear using materials, patterns, colours and fastenings. While this approach may not fit a primary pair of day‐to‐day therapeutic footwear for all patients, it could be a very useful solution in the design of specific therapeutic footwear for specific formal social occasions or for patients who are more willing to showcase their aesthetically pleasing therapeutic footwear in day‐to‐day life [60]. Visual illusions can be used to give the impression of a more pointed toe box and different design features (colours, thinner Velcros and smoother shoe shapes) can be used to make the footwear appear more aesthetically appealing. One must take into account though, that therapeutic footwear will always be shaped in line with the foot. With foot deformities frequently present, it may not always be possible to find a solution that is satisfactory from both a protective and aesthetic perspective. While having multiple pairs for different occasions may facilitate most opportunities, this is often not (financially) feasible. Regardless of the situation, it is important to discuss the issue with the patient beforehand. The aim of such a discussion is to find the best possible compromise between protective footwear features to prevent foot ulcers and aesthetic footwear features as an item of clothing to in turn improve adherence [11].

### 3.5 Waterproof Therapeutic Footwear

As regular therapeutic footwear is not designed for use in water, patients may instead walk barefoot exposing the feet to both acute and chronic trauma. Gumboots and special ‘water shoes’ are available for the general population to protect the feet from acute trauma at the beach and in the water, but one study reported that they may actually cause foot ulcers [35], and they do not incorporate features to reduce plantar pressures. Thus, when a patient plans to spend much time standing or walking in water‐related activities, or has deformities that increase plantar pressures, standard water shoes may not be appropriate, and special waterproof therapeutic footwear designed for use in water is needed. Such footwear needs to be developed.

### 3.6 Protective Hosiery and Other Non‐Footwear Solutions

In certain contexts, the most effective and acceptable way to protect the foot from trauma is not via footwear itself, but from other strategies to protect the foot. In these cases, clinicians need to ‘think outside the (shoe) box’ to identify the most threatening trauma to protect against in the specific context, and to identify the most fitting solution that protects the foot and is acceptable for the patient.

Protective hosiery may be more acceptable to patients not used to wearing footwear indoors, as hosiery is lightweight and feels more like wearing socks. For patients who find it inconvenient to put footwear on at night when visiting the bathroom, sleeping with protective hosiery may be an alternative, eliminating the need to don footwear at night. In addition, hosiery is not perceived as footwear and may be more readily accepted in places‐of‐worship and at home. Older studies on protective hosiery found modest reductions of plantar pressures [61], but more recent studies on other hosiery designs have demonstrated more effective plantar pressure reduction [62]. Another interesting approach is to let the patient use a custom‐made insole inside the sock to reduce plantar pressures [63]. These innovations may protect the foot against high plantar pressures and plantar burns.

Also, reducing risks is not only a matter of footwear and other devices, but also about discussing with patients the different contexts and how to modify or dose their weight‐bearing activities, such as daily steps, to not surpass the threshold of what the tissues can tolerate [64]. For example, if a patient wishes to use tight‐fitting high‐heeled footwear on a social occasion, the patient and clinician may compromise and agree that the patient should remove the high‐heeled footwear when sitting down and otherwise limit walking or dancing during the event to prevent foot ulcers. Being proactive in discussions about contexts that imply ulceration risks may be extra important in relation to holiday plans of patients; before they go, enquire about the types of activities they plan to engage in, what type of footwear they plan to use in each different context, and how to act if (pre‐) ulcers develop [35, 40, 47]. Development is ongoing of remote patient monitoring systems with sensors measuring plantar pressures, foot temperatures, footwear adherence, and weight‐bearing activity, which may further help clinicians to give person‐centred advice to reduce risk of foot ulcerations [65].

## 4 Discussion

The aim of this paper was to propose and discuss a contextual approach to considering therapeutic footwear solutions. Seven contexts that are particularly challenging for using therapeutic footwear were discussed and six different footwear solutions proposed. Although therapeutic footwear would in theory provide the best protection in most of these contexts, using it is often not realistic because of the contextual demands that conflict with its use. Instead of insisting that the patient should adhere to the ‘one‐(therapeutic) shoe‐fits‐all’ solution and use the same therapeutic footwear across multiple different contexts, it may be more fruitful to be upfront and identify the more challenging contexts in which footwear is used with the patient to avoid ‘strategic non‐adherence’, where the patient may deliberately choose to not use recommended footwear to live a more normal daily life [18].

However, this contextual approach may come with a risk of normalising suboptimal protection, that is, prescribing footwear that provides less than optimal protection when patient education could have resulted in the patient accepting more effective footwear solutions. For this reason, we suggest a two‐step process. First, clinicians should discuss with the patient what their risks are and what would be the ideal footwear solutions to prescribe to protect their feet in each context, given financial and organisational constraints. A single education session using a structured model [5] has shown to improve wearing of footwear by 1 hour in the weeks following in people with low adherence [55]. If the patient accepts these ideal footwear solutions, they should be prescribed. However, as a second step, if the patient despite discussion and education on risks and benefits does not accept one or more of these solutions, the clinician and patient should discuss how to design footwear solutions that are both acceptable to the patient and offer some (the most) protection for the feet. A contextual approach thus requires a shift of focus; from convincing patients to use one pair of therapeutic footwear in all contexts to instead minimising risks in each context [66]. This focus shift requires the clinician to have open discussions with the patient about their personal contexts and circumstances, to together find solutions that offer protection for the feet and are acceptable for the patient. When patients feel they are being heard and understood, they are more likely to adhere to using their footwear [67] and better communication also allows discussing expectations and acceptance of the footwear—both contributors to adherence [68]. In some cases, this contextual approach may result in a trade‐off for less protection than optimal, such as if using protective hosiery in the house instead of therapeutic footwear. It is then important that the patient understands that risks of foot ulceration increase when a less‐than‐optimal footwear solution is chosen, especially when the ulcer risk is high due to severe foot deformities or peripheral artery disease.

Potential barriers to a contextual approach to therapeutic footwear are increased footwear costs and constraints in footwear reimbursements. Given that patients wish to use different types of footwear in different physical and sociocultural settings, more than one pair of therapeutic footwear may be needed to protect the feet [66]. This will increase footwear costs, especially as many of the footwear solutions needed are currently only available with custom‐made footwear. However, given the large and growing population of people with diabetes, there appears a large enough market to develop more diverse prefabricated therapeutic footwear solutions for different contexts. In addition, the number of footwear pairs needed is restricted by the fact that certain footwear solutions are useful in more than one context (Table 2) and footwear costs should be considered in relation to the costs of avoiding ulcers that may have developed because of low adherence to using appropriate footwear in certain contexts. Assuming a treatment cost of 10,000 Euros per foot ulcer episode [69] and 400 Euros per pair of custom‐made therapeutic footwear, the cost of a single ulcer episode may equal 25 pairs of therapeutic footwear. Footwear costs are an even bigger issue in low‐ and middle‐income countries, where 75% of people across the world with diabetes live [51] and costs related to foot ulcers put a significant burden on patients and their families [37]. Therefore, there is a pressing need for footwear manufacturers to develop low‐cost prefabricated therapeutic footwear that is economically accessible to patients living in low‐ and middle‐income countries. There is also a need for cost‐effectiveness studies on therapeutic footwear and other solutions to prevent foot ulcers.

We recommend future studies should investigate a contextual approach to therapeutic footwear. Only a few studies have differentiated between different contexts, and all have had a rather narrow focus. Five studies separately assessed adherence in the home and away from home [14, 19, 24, 25, 26], and one study asked patients for their perceived benefits of therapeutic footwear at home and at work [70]. Future studies need to assess adherence levels, perceived benefits and effectiveness, in a wider range of contexts. Furthermore, almost all footwear adherence research has been conducted in Europe and North America [13, 16, 71], where resources are relatively large, climates often mild or cold, and footwear habits may differ from many other parts of the world. Thus, there is a need for assessing adherence and other aspects of footwear use in other countries, cultures and climates. Also, there is a need for more development and research on how to improve footwear adherence, both generally and in different and specified contexts. This may include development of innovative footwear solutions, such as, different lightweight, cooler, easy‐to‐apply, safety and waterproof therapeutic footwear options and even non‐footwear options, such as protective hosiery and activity dosing. Furthermore, there is a need for studies on clinical effectiveness and cost‐effectiveness of these solutions.

## 5 Conclusions

A contextual approach to therapeutic footwear for people at risk of foot ulcers may lead to a more constructive and personalised approach in patient care. We identified seven challenging footwear contexts that are currently not well addressed, and six potential solutions that may address them better. These should be the focus of future research and development with the aim to improve patient adherence and reduce foot ulceration rates.

## Author Contributions

**Gustav Jarl:** conceptualization, visualization, writing – original draft, writing – review editing. **Peter A. Lazzarini:** conceptualization, writing – review editing. **Gulapar Srisawasdi:** writing – review editing. **Jaap J. van Netten:** conceptualization, writing – review editing.

## Funding

The authors have nothing to report.

## Ethics Statement

The authors have nothing to report.

## Consent

The authors have nothing to report.

## Conflicts of Interest

The authors declare no conflicts of interest.

## Permission to Reproduce Material From Other Sources

The authors have nothing to report.

## Data Availability

Data sharing not applicable to this article as no datasets were generated or analysed during the current study.
